# The Influence of Screw Positioning on Cage Subsidence in Patients with Oblique Lumbar Interbody Fusion Combined with Anterolateral Fixation

**DOI:** 10.1111/os.13882

**Published:** 2023-09-28

**Authors:** Kai Wang, Xiandi Wang, Zhuhai Li, Tianhang Xie, Lihang Wang, Chuan Luo, Shishu Huang, Jiancheng Zeng

**Affiliations:** ^1^ Department of Orthopaedic Surgery and Orthopaedic Research Institute, West China Hospital Sichuan University Chengdu China; ^2^ Department of Spine Surgery The People's Hospital of Guangxi Zhuang Autonomous Region Nanning China; ^3^ Department of Spine Surgery Guizhou Provincial Orthopedics Hospital Guiyang China; ^4^ School of Mechanical Engineering Sichuan University Chengdu China

**Keywords:** Cage Subsidence, Oblique Lumbar Interbody Fusion, Screw Position, Screw Trajectory

## Abstract

**Objectives:**

Cage subsidence (CS) has been reported to be one of the most common complications following oblique lumbar interbody fusion (OLIF). To reduce the incidence of CS and improve intervertebral fusion rates, anterolateral fixation (AF) has been gradually proposed. However, the incidence of CS in patients with oblique lumbar interbody fusion combined with anterolateral fixation (OLIF‐AF) is still controversial. Additionally, there is a lack of consensus regarding the optimal placement of screws for OLIF‐AF, and the impact of screw placement on the incidence of CS has yet to be thoroughly investigated and validated. The objective of this investigation was to examine the correlation between screw placements and CS and to establish an optimized approach for implantation in OLIF‐AF.

**Methods:**

A retrospective cohort study was undertaken. From October 2017 to December 2020, a total of 103 patients who received L4/5 OLIF‐AF for lumbar spinal stenosis or spondylolisthesis or degenerative instability in our department were followed up for more than 12 months. Demographic and radiographic data of these patients were collected. Additionally, screw placement related parameters, including trajectory and position, were measured by anterior–posterior X‐ray and axial CT. Analysis was done by chi‐square, independent t‐test, univariable and multivariable binary logistic regression to explore the correlation between screw placements and CS. Finally, the receiver operating characteristic (ROC) curve analysis was used to evaluate the predictive ability of screw placement‐related parameters.

**Results:**

A total of 103 patients were included, and CS was found in 28 (27.18%) patients. Univariable analysis was firstly performed for each parameter. Next, variables with *p*‐value of <0.05, including bone mineral density (BMD), concave morphology, and screw placement‐related parameters were included in the multivariate logistic regression analysis. Significant predictor factors for subsidence were coronal plane angle (CPA) (OR 0.580 ± 0.208, 95% CI 1.187–2.684), implantation point (IP) (L4) (OR 5.732 ± 2.737, 95% CI 1.445–12.166), and IP (L5) (OR 7.160 ± 3.480, 95% CI 1.405–28.683). Furthermore, ROC curves showed that the predictive accuracy of CS was 88.1% for CPA, 77.6% for IP (L4) and 80.9% for IP (L5).

**Conclusions:**

We demonstrate that the trajectory of vertebral screws, including angle and position, was closely related to CS. Inserting screws parallel to each other and as close to the endplate as possible while keeping the cage inside the range of the superior and inferior screws are an optimal implantation strategy for OLIF‐AF.

## Graphical abstract

We demonstrate that the trajectory of vertebral screws, including angle and position, was closely related to CS. Inserting screws parallel to each other and as close to the endplate as possible while keeping the cage inside the range of the superior and inferior screws are an optimal strategy for OLIF‐AF.

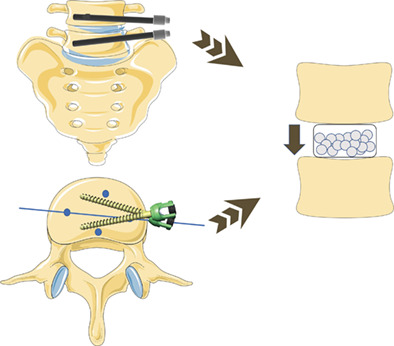

## Introduction

Lumbar interbody fusion (LIF) combined with bone grafting and various types of cages is regarded as an effective treatment for patients with degenerative lumbar diseases.^1^ Different from the direct decompression of other posterior LIF procedures, oblique lumbar interbody fusion (OLIF) is a novel indirect decompression and has been highlighted recently.^2^ The principle of indirect decompression is to insert a large cage to increase the intervertebral height, enlarge the intervertebral foramen, unbuckle ligamentum flavum, and restore the protruding intervertebral disc. These factors enhance the volume of the spinal canal and eventually stabilize the surgical segment. Additionally, OLIF does not destroy paravertebral muscles and ligaments, which has the advantages of less iatrogenic trauma and faster recovery.^3^, ^4^ However, postoperative cage subsidence (CS) has always been a major problem that haunts the surgeons, and the incidence of CS has been reported to range from 10% to 45%.^5^, ^6^, ^7^, ^8^ In the case of subsidence, the vertebral body may be fused inappropriately, leading to further complications and increasing pain and cost to the patients. To avoid such complications, supplemental fixation has been proposed. Finite element analysis has shown that supplemental fixation could reduce the maximum stresses on the endplate in OLIF compared with OLIF stand‐alone.^9^ Posterior pedicle screw fixation (PSF) is preferred by most spinal surgeons, Traditionally, the patient is repositioned in the prone position for the pedicle screw fixation after the lateral position, which is undoubtedly increased extra incision and surgical exposure to the posterior spinal area. Therefore, some novel techniques were proposed in which the pedicle screws are placed while the patient remains in the lateral position following the LLIF, including PTP technique^10^ or by posterior percutaneous pedicle fixation with robotic or O‐arm assistance.^11^ However, operators are less accustomed to the lateral screw placement, the learning curve remains steep, and few institutions have installed these instruments because of the cost.^12^, ^13^ To overcome the disadvantages of PF, an anterolateral *in situ* screw‐rod system was proposed as a supplemental fixation for OLIF. In addition, our previous clinical studies have proven that oblique lumbar interbody fusion combined with anterolateral fixation (OLIF‐AF) significantly reduced the incidence of CS compared with the OLIF stand‐alone procedure.^5^, ^14^ As reported, risk factors related to CS involving OLIF are bone mineral density (BMD), endplate morphology, and cage parameters.^15^ Although numerous risk factors above have been investigated, few have evaluated the correlation between the CS and supplemental fixation.

To date, most studies have found that insertional screw positions affect surgical segment stiffness and local stress distribution, which are closely associated with the incidence of CS.^16^ Newcomb et al. suggested that angulations in both the sagittal and axial planes affected fixation strength during posterior spine fixation.^17^ For OLIF‐AF, screw placement is not restricted by the pedicle; thus, theoretically, anterolateral screws can be placed in different trajectories. However, to date, related studies have only focused on the influence of different fixation methods on the incidence of CS. No study focused on the effects of screw trajectory and position on the incidence of CS. Additional, there is no consensus on the placement of screws for OLIF‐AF. Consequently, we assume that local biomechanical behavior is responsible for the CS. Specifically, the aims of this study were: (1) to determine and validate the established risk factors for CS, including demographic and radiological data; (2) to examine the correlation between screw placement related parameters, including trajectory and position, and CS; (3) to better guide the clinical application of OLIF‐AF surgery and might thereby help improve treatment results and minimize complications.

## Materials and Methods

### Study Design

This was a retrospective cohort study and was approved by the Ethics Committee of the West China Hospital, Sichuan University(Ethics approval number: 2020‐554). Radiographic data of OLIF‐AF patients in our hospital were retrieved and screened for eligibility, including X‐ray, CT, and MRI. Clinical data included sex, age, preoperative diagnosis, length of stay (LOS), follow‐up time, cage parameters (cage height and cage length). The inclusion criteria were as follows: (1) mild or moderate spinal stenosis (Schizas grade A or B^18^) or mild degenerative lumbar spondylolisthesis (Meyerding grade I and II^19^) or degenerative instability at L4‐5 (the vertebral slippage was ≥4 mm or if the mobility in flexion or extension was ≥10°^20^); (2) a follow‐up period of at least 12 months; (3) lumbar CT and X‐ray radiographies before surgery, 1 day after surgery, and 12 months after surgery; and (4) no spinal surgery history. The exclusion criteria were as follows: (1) Patients who underwent multilevel surgery; (2) Patients with infectious or neoplastic conditions; (3) Patients who were diagnosed with severe stenosis (Schizas grade C or D) or stenosis caused by calcifed disc or bony spur formation; (4) Patients who were diagnosed with isthmic spondylolisthesis or severe degenerative spondylolisthesis (Meyerding grade III‐IV); (5) Patients who had follow‐up of less than 12 months. Two of the authors independently completed the measurements and the mean values were collected for analysis.

### OLIF Procedures

Detailed descriptions of the OLIF‐AF procedure have been included in our previous study.^5^ Briefly, patients were placed in a right decubitus position under general anesthesia. A 5‐cm skin incision was made in the lateral abdominal region, parallel to the iliac crest, followed by dissections of the abdominal muscles, including the external oblique, the internal oblique, and the transverse abdominis, along the directions of the corresponding muscle fibers using a muscle‐splitting approach. The retroperitoneal space was accessed by blunt dissection, after which the peritoneal content was mobilized anteriorly. The psoas muscle was identified and retracted posteriorly, after which the sympathetic chain and ureter were mobilized anteriorly. Subsequently, the left anterolateral side of the L4‐5 intervertebral disc was exposed. C‐arm fluoroscopy was performed to confirm the proper spinal level, and the tubular retractor system was docked. Furthermore, discectomy was performed, and the endplate was prepared. Then, a polyetheretherketone (PEEK) cage (Clydesdale, Medtronic, USA) (lordotic angle: 6°) filled with artificial bone was inserted vertically into the intervertebral space. Finally, two vertebral screws were inserted close to the endplate on the lateral sides of the L4 and L5 vertebral bodies and connected with a titanium rod.

### Radiographic Evaluation

**Fig. 1:**
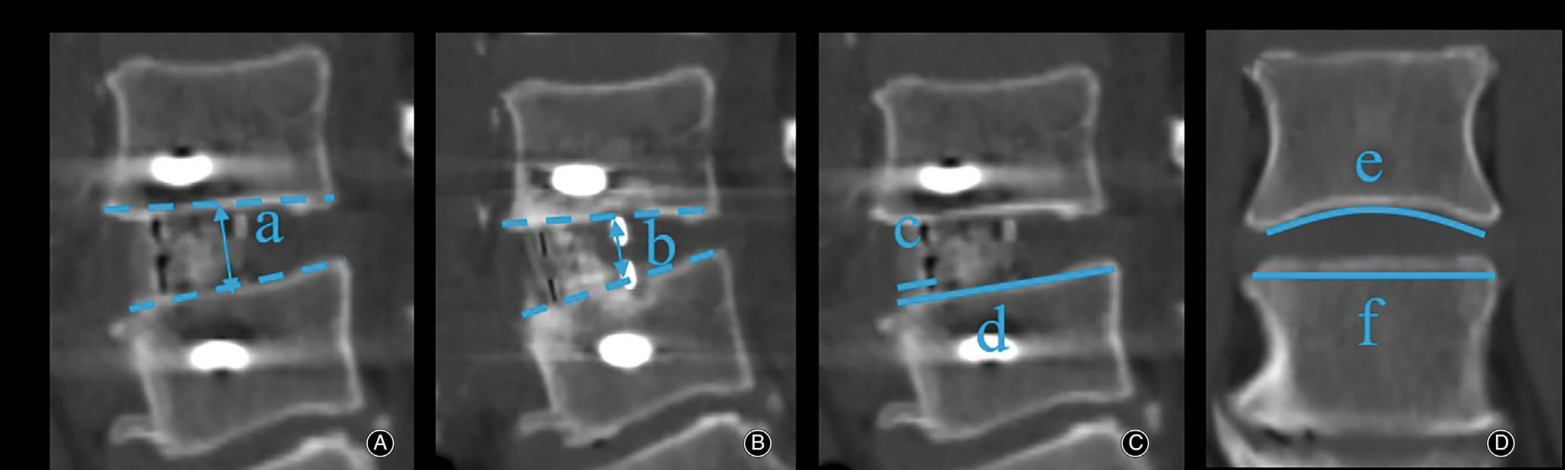
Measurement of disc height on 2D CT midsagittal plain reconstruction. (a, b) The vertical distance between the midpoint of the superior and inferior endplates; (c) the distance between the anterior marker of the cage and the anterior edge of the vertebral body; (d) the distance between the anterior edge of the vertebral body and the posterior edge of the vertebral body; (e) concave endplate morphology; (f) flatendplate morphology; (A) disk height measured 1 day postoperatively; (B) disk height measured at the last follow‐up; (C) cage position; (D) endplate morphology.

**Fig. 2:**
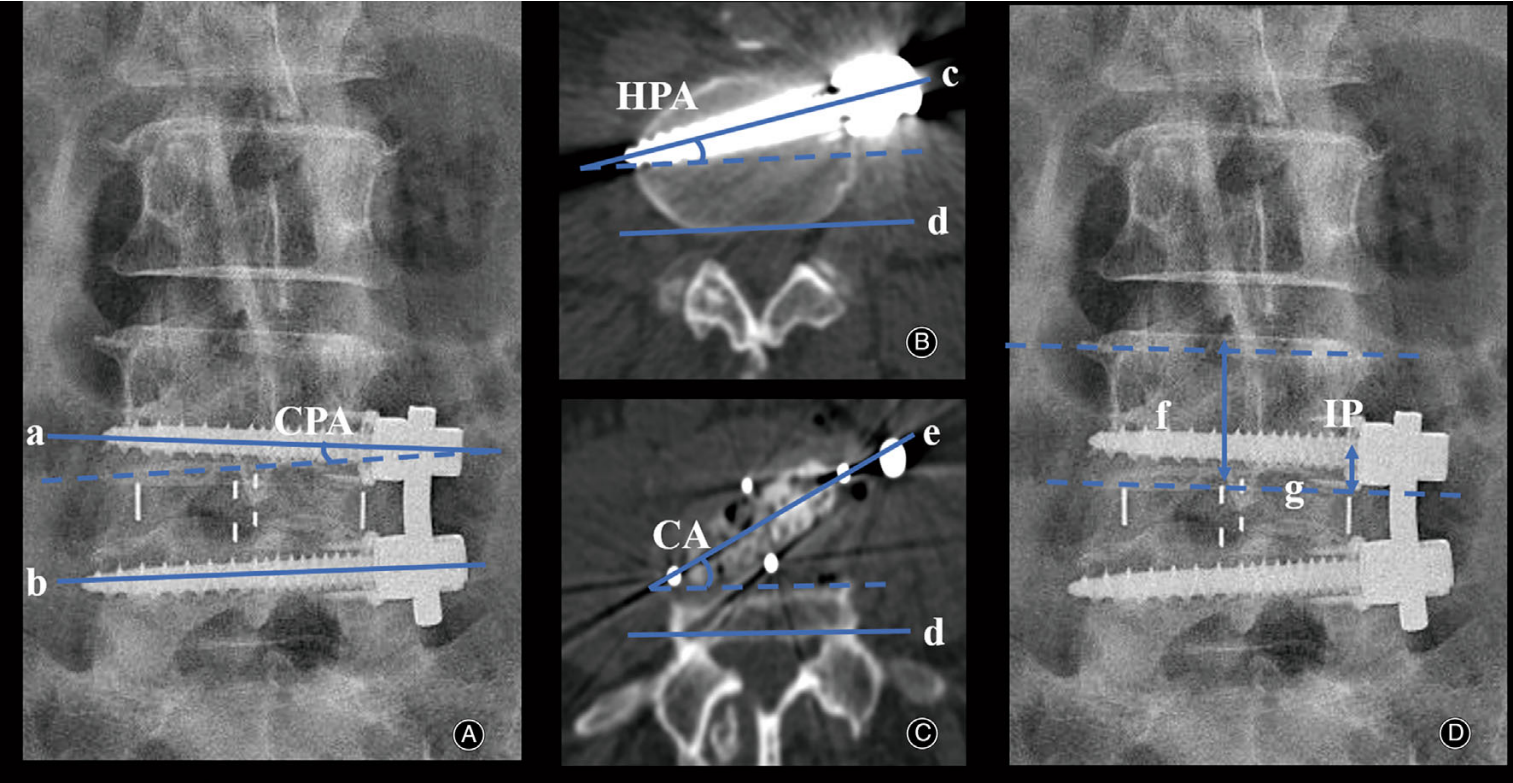
Measurements of radiological parameters related to the vertebral screw measurements. (a) The long axis of the upper vertebral screw; (b) the long axis of the lower vertebral screw; (c) the long axis of the screw; (d) the posterior edge of the vertebral body; (e) the long axis of the cage; (f) the height of the entire vertebral body; (g) the distance between the contact point and the endplate. (A) CPA measured by antero‐posterior X‐ray; (B) CA measured by using axial CT; (C) SA measured by using axial CT; (D) IP measured by antero‐posterior X‐ray.

**Fig. 3:**
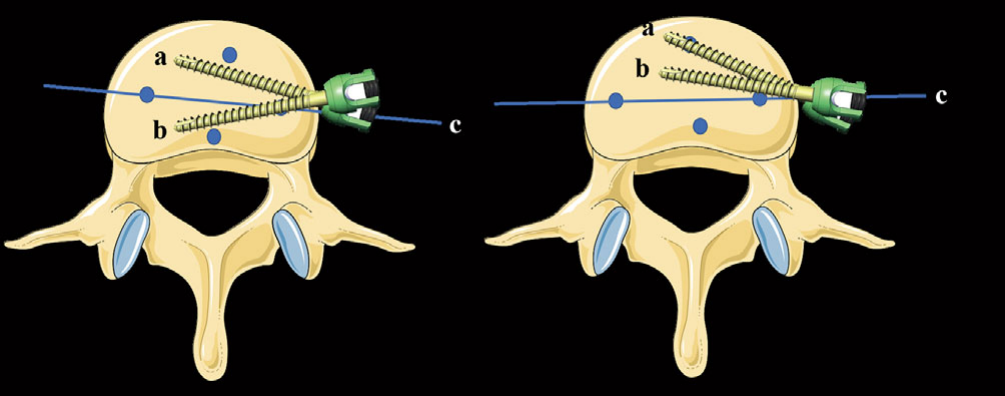
Schematic diagram of the position relationship between SSA, ISA, and CA. (a) The long axis of the upper vertebral screw; (b) the long axis of the lower vertebral screw; (c) the long axis of the cage. (A) The value of AC is within the range of SSA and iSA; (B) The value of AC is outside of the range of SSA and ISA.

**Fig. 4:**
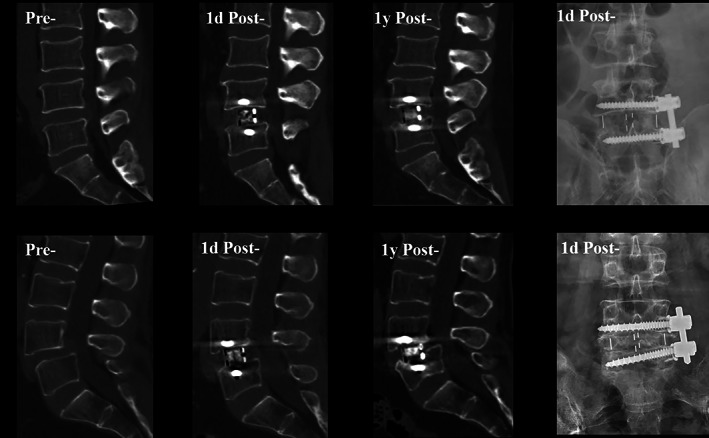
The two typical cases of patients who underwent L4‐5 OLIF‐AF and their radiological outcomes before surgery as well as at 1 day, 1 year after that.

All patients underwent lumbar 2D‐CT and X‐ray examinations before surgery, 1 day after surgery, and 1, 3, 6, and 12 months after surgery. Disc height (DH) was measured as the vertical distance between the midpoint of the superior and inferior endplates on 2D CT midsagittal plain reconstruction^21^, ^22^ (Figure 1A,B). BMD was expressed by the minimum T score obtained from the hip using dual‐energy X‐ray absorptiometry (DEXA) scans performed preoperatively. Moreover, data related to cage position was collected. CS was defined as a 2 mm or more reduction in DH at the last follow‐up compared with 1 day postoperatively by the median sagittal plane of CT.^22^ Cage position was defined by the percentage of the distance between the anterior marker of the cage and the anterior edge of the vertebral body (Figure 1C).^23^ The endplate morphology was divided into flat type and concave one (Figure 1D).^23^, ^24^ Screw placement‐related parameters include trajectory and position, and the radiological parameters related to the trajectory and position of vertebral screws can be divided into two angles and one point. The screw trajectory consists of coronal plane angle (CPA) and horizontal plane angle (HPA). The former refers to the coronal plane angle between the long axis of the superior and inferior vertebral screws by using antero‐posterior X‐ray (Figure 2A), while the horizontal plane angle between the long axis of screw and the posterior edge of the vertebral body was recorded as the latter by using axial CT^25^, ^26^ (Figure 2B), namely, the superior screw angle (SSA) and inferior screw angle (ISA). In addition, the horizontal plane angle between the long axis of the cage and the posterior edge of the vertebral body was defined as cage angle (CA) to replenish the axial parameters^26^ (Figure 2C). Notably, the trajectory of vertebral screws is three‐dimensional, selection of a single coronal CT layer cannot avoid the interference of sagittal interference, and the appropriate plane could not be selected. Therefore, X‐ray was used for the coronal parameters after consulting relevant literature. A schematic diagram depicting the position relationship between the SSA, ISA, and CA are shown in Figure 3 to illustrate the definition. Finally, the point refers to the screw implantation point (IP), which was quantified by calculating the ratio of the distance between the contact point and the endplate to the height of the entire vertebral body on anterior–posterior X‐ray (Figure 2D). For a better interpretation of the purpose of our study, two typical cases of OLIF‐AF were shown in Figure 4. Notably, two of the authors independently completed the measurements and the mean values were collected for analysis.

### Statistical Analysis

SPSS 26.0 (IBM Corp., Armonk, New York, USA) software was used for statistical evaluation. Data are expressed as the mean ± standard deviation (SD) for continuous variables and number for categorical variables. Inter‐observer reliability between the two observers scoring the videos continuously was assessed by intraclass correlation coefficients (ICC). Two independent samples t tests were performed for continuous variables, and chi‐squared analysis was performed for categorical variables. Significance was set at *p* < 0.05. Subsequently, binary logistic regression analysis was performed after selecting the significant risk factors using univariate logistic regression. Moreover, receiver operating characteristic (ROC) analysis was conducted to establish the threshold between subsidence and non‐subsidence to further evaluate the predictive ability of screw placement‐related parameters. Finally, the area under the curve and the most appropriate threshold (cutoff value) corresponding to the screw parameters with a higher sensitivity and specificity were calculated.

## Results

### Baseline Demographic Data

A total of 103 patients were enrolled in the study between October 2017 and December 2020 in our hospital. The baseline demographic data are shown in Table 1.There were 40 males and 63 females with a mean age of 60.66 ± 8.09 years at surgery. CS was found in 28 (27.18%) patients. Patients with CS had a lower average DEXA T‐score (−2.03 ± 0.53 vs. −1.73 ± 0.63, *p* = 0.026). In contrast, there were no significant differences in age (*p* = 0.0.881), sex (*p* = 0.691), BMI (*p* = 0.699), LOS (*p* = 0.872), follow‐up time (*p* = 0.695), diagnosis (*p* = 0.765), cage height (*p* = 0.709), or cage length (*p* = 0.118) between patients with or without CS.

**TABLE 1:** Demographic variables and radiographic parameters of both groups.

Variable	Both cohorts	Non‐subsidence	Subsidence	*p* value
Patients	103	75	28	
Age (years)	60.66 ± 8.09	60.58 ± 8.14	60.85 ± 8.09	0.881
Sex				0.691
Male	40	30	10	
Female	63	45	18	
BMI (kg/m^2^)				0.699
≥25	45	24	21	
<25	48	37	21	
BMD	−1.81 ± 0.62	−1.73 ± 0.63	−2.03 ± 0.53	**0.026**
LOS (days)	6.00 ± 1.60	6.09 ± 1.43	6.03 ± 2.00	0.872
Follow‐up time (months)		16.78 ± 6.28	17.32 ± 5.72	0.695
Diagnosis				0.765
Lumbar spinal stenosis	63	33	30	
Lumbar spondylolisthesis	26	12	14	
Lumbar instability	14	9	5	
Cage height (mm)	12.66 ± 1.46	12.69 ± 1.45	12.57 ± 1.52	0.709
Cage length (mm)	51.21 ± 3.39	51.53 ± 3.28	50.35 ± 3.58	0.118

*Note*: Data presented as mean ± standard deviation.

Abbreviations: BMI, body mass index; LOS, length of stay.

### Radiological Results

The analysis of subsidence in endplates at varying insertional screw angles and positions is presented in Table 2. The ICC results suggested that the measurement indexes of the two readers were consistent (all ICC >0.75, *p* < 0.05; in Table SS1), and the mean values were collected for analysis. In terms of angle, the CPA was 3.46 ± 2.40 in non‐subsidence and 7.57 ± 2.47 in subsidence (*p* < 0.001). From the horizontal plane, when the value of CA is within the range between the SSA and ISA, the incidence of CS is significantly lower (*p* < 0.001). In terms of position, IP was similarly strongly associated with the incidence of CS (*p* < 0.001). The distance between IP(L4) and endplate was 0.37 ± 0.13 in non‐subsidence and 0.53 ± 0.15 in subsidence group, the distance between IP(L5) and endplate was 0.38 ± 0.13 in non‐subsidence and 0.54 ± 0.12 in subsidence group. Additional, patients with CS had a more concave morphology of inferior endplate (36.36% vs. 7.69%, *p* = 0.031) and superior endplate (66.66% vs. 16.66%, *p* = 0.014). However, the trajectory angle of the superior and inferior vertebral screw (SSA, ISA) and cage (CA) alone, cage position, and fusion rate, did not correlate significantly with CS (SSA: *p* = 0.084; ISA: *p* = 0.795; CA: *p* = 0.986; cage position: *p* = 0.389; fusion rate: *p* = 0.354).

**TABLE 2:** Radiographic parameters of both groups.

Variable	Both cohorts	Non‐subsidence	Subsidence	*p* value
Cage position (mm)	21.36 ± 3.66	21.56 ± 3.67	20.85 ± 3.65	0.389
Superior endplate morphology (Flat/Concave)	22/81	20/55	2/26	**0.031**
Inferior endplate morphology (Flat/Concave)	34/69	30/45	4/24	**0.014**
Fusion rate	29/103	23/75	6/28	0.354
CPA (°)	4.58 ± 3.03	3.46 ± 2.40	7.57 ± 2.47	**<0.001**
SSA (L4) (°)	7.41 ± 5.14	7. 05 ± 5.13	8.38 ± 5.13	0.536
ISA (L5) (°)	11.43 ± 6.00	11.55 ± 5.68	11.12 ± 6.91	0.104
CA (°)	9.78 ± 5.94	10.07 ± 5.80	9.00 ± 6.33	0.745
Whether the value of CA is within the range between the SSA (L4) and ISA (L5)				**<0.001**
Yes	53	51	2	
No	50	24	26	
IP (L4) (mm)	0.41 ± 0.15	0.37 ± 0.13	0.53 ± 0.15	**<0.001**
IP (L5) (mm)	0.42 ± 0.15	0.38 ± 0.13	0.54 ± 0.12	**<0.001**

*Note*: Data presented as mean ± standard deviation.

Abbreviations: CA, cage angle; CPA, coronal plane angle; IP, implantation point; ISA, inferior screw angle; SSA, superoior screw angle.

Next, we performed a multivariable analysis by including variables with statistically significant results in univariable analysis into a multivariable model (Table 3). Notably, significant predictor factors for subsidence were CPA (OR 0.580 ± 0.208, 95% CI 1.187–2.684), IP(L4) (OR 5.732 ± 2.737, 95% CI 1.445–12.166), and IP(L5) (OR 7.160 ± 3.480, 95% CI 1.405–28.683).

**TABLE 3:** Multivariable analysis of statistically significant variables.

Factor	OR	95%CI		*p* value
BMD	−1.127 ± 0.751	0.074	1.413	0.134
Superior endplate morphology (Flat/Concave)	1.548 ± 1.359	0.328	67.476	0.255
Inferior endplate morphology (Flat/Concave)	0.434 ± 1.189	0.150	15.854	0.715
CPA (°)	0.580 ± 0.208	1.187	2.684	**0.005**
Whether the value of CA is within the range between the USA (L4) and LSA (L5)	2.254 ± 0.978	1.401	64.756	**0.021**
SIP (L4) (mm)	5.732 ± 2.737	1.445	12.166	**0.036**
SIP (L5) (mm)	7.160 ± 3.480	1.405	28.683	**0.040**

**Fig. 5:**
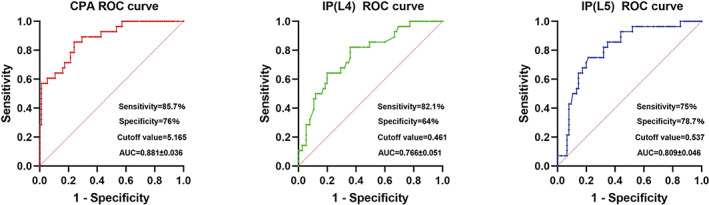
ROC analysis of CPA in red, IP(L4) in green and IP(L5) in blue, with respective sensitivity, specificity, cutoff, and AUC shown.

Further, ROC curves were drawn to evaluate the predictive ability of screw placement‐related parameters (Figure 5). The area under the curve (AUC) of CPA was 0.881 (95% confidence interval [CI]: 0.810–0.952). The best threshold for predicting CPA was 5.16 (sensitivity: 85.7%, specificity: 76%). The AUC of IP(L4) was 0.766 (95% CI: 0.666–0.867), and the most appropriate threshold of IP(L4) was 0.46 (sensitivity: 82.1%, specificity: 64%). The AUC of IP(L5) was 0.809 (95% confidence interval CI: 0.719–0.899), and the most appropriate threshold of IP(L5) was 0.41 (sensitivity: 75%, specificity: 78.7%).

## Discussion

The principal finding of the present study is that we found the trajectory of vertebral screws, including angle and position, was independently associated with an increased risk of CS and these variables could independently predict the postoperative complication.

The incidence of CS has been reported to range from 10% to 45%.^5^, ^6^, ^7^, ^8^ In our research, CS was observed in 27.18% of the participants. This relatively lower subsidence rate in our study may be attributed to the anterolateral fixation. Our previous study reported that sufficient stability for fusion can be obtained by combination with AF.^27^ Similar findings were noted in a study performed by Ha et al. They found no significant differences in the ODI score, fusion rate, or incidence of complications between OLIF+AF and OLIF+PF.^28^ Therefore, anterolateral fixation could be an effective adjunct to prevention of the postoperative complication.

In this study, we first verified that patients with CS had a lower BMD and a more concave endplate morphology than those without CS, which was similar to previous studies.^15^ Lower BMD is well‐established risk factor for bone for CS. From a biomechanical perspective, low BMD triggers stress concentration in surgical segment and increases the risk of a poor local biomechanical environment in OLIF patients. Therefore, anti‐osteoporosis treatment should be considered an effective therapy to avoid subsidence risk.^29^, ^30^ Additional, previous studies confirmed that relatively flat endplates significantly diminish the incidence of subsidence. Consequently, the morphology of cage surface should be also taken into consideration when designing the next generation of cage,^23^, ^24^ which is also the future research direction of prevention of CS.

Secondly, the results showed that anterior instrumentation should be placed as close to the endplate as possible, which may be attributed to the trabecular structure of the spine. Higher local stress between the cage and endplate is the direct cause of CS. After additional anterolateral fixation, the stresses can be shared by nail‐stick system to avoid stress concentrations. In fact, many parameters affect the biomechanical performance of the instrumentation,^31^, ^32^, ^33^ of which the trajectory of the implanted screws is the most important. The relationships between the trajectory of the implanted screws and the mechanical environments have been widely reported in different fusion fixation methods.^34^ Previous studies have found that the bone quality near the endplate is better, which can better hold the screw and share the stress on the endplate.^35^, ^36^ In addition, Zhu et al. suggested that the screw penetrated the cortex of the opposite vertebral body, which was consistent with our usual implantation method.^37^ In fact, when the screw was inserted from the middle of one side of the vertebral body and penetrated the cortex from the middle of the contralateral vertebral body, rigid tricortical fixation was formed due to the trajectory close to the endplate, which could both provide better initial stiffness under all loading conditions and provide superior protective effects on the interbody cage and screw‐bone interface under lateral‐bending conditions. The technique of tri‐cortical pedicle screw (TCPS) has been used to improve the anchoring strength in the vertebrae.^37^ In practice, the technique of TCPS can be applied to enhance the grip strength of the screws and reduce the risk of CS.

Finally, our study showed that the odds of CS will increase when the angle between the superior and inferior vertebral screws is too large in the coronal plane. Several studies reached a similar conclusion to that of our study. Luc et al. suggested that anterior instrumentation should be put in place with the axis of the screws aligned as close as possible with the coronal plane.^38^ This phenomenon may be partly due to nonuniform stress distribution, which will result in an inability to share more stress on the endplate. For the horizontal plane, we found that CS is more likely to occur when the cage is located outside the upper and lower screws. Interestingly, there is no correlation with the angle of the screw and CS in the horizontal plane when ignoring the positional parameters of the cage. This result may be due to lateral screws inserted in the upper and lower vertebral body sharing the stress in the vertical plane of the contact surface between the cage and the endplate, forming significant shear strains. However, further cadaver experiments and more biomechanical analyses are necessary to confirm our findings, which will be the focus of our next studies. Notably, accurate insertion of the screw trajectory is technically difficult in clinical practice, which requires considerable experience and mastery of skills. With the advent of robot‐assisted orthopaedic surgery, robot‐assisted pedicle screw fixation had several benefits, including capability of preoperative planning, high accuracy of screw insertion, and low radiation exposure. Numbers of studies showed robot‐assisted pedicle screw placement is more accurate than the conventional method after analyzing the trajectory of screw insertions.^39^, ^40^ Therefore, the integration of robot‐aided screw fixation and screw trajectory planning a future prospect to establish an optimized approach for implantation in OLIF‐AF.

### Limitations

Some limitations of this study should not be ignored. This was a retrospective, single‐center study with relatively limited samples that might be insufficient for detecting significant differences between subgroups, which need to be validated by prospective studies. Additionally, we only selected patients who underwent single‐level L4‐5 surgery, which may not be appropriate for others, including multiple levels. Although we found interesting correlations between these factors, we did not use experimental methods such as animal experiments and additional cadaver testing to evaluate the biomechanics of the screw fixation system, which is the focus of our subsequent research to understand the underlying mechanism.

### Prospects of Clinical Application

Inserting screws parallel to each other and as close to the endplate as possible while keeping the cage inside the range of the superior and inferior screws are an optimal implantation strategy for OLIF‐AF. In practice, the technique of TCPS can be applied to enhance the grip strength of the screws and reduce the risk of CS.

## Conclusions

We demonstrated that the trajectory of vertebral screws, including angle and position, was closely related to CS. It may be more helpful to prevent CS by inserting placement of screws parallel in the coronal plane as close to the endplate as possible while keeping the CA within the range of the SSA and ISA in the horizontal plane. This implantation method conforms to the principle of the TCPS technique. In conclusion, our findings provide practical reference values for reducing the occurrence of postoperative CS and the clinical application of screw placement for OLIF‐AF.

## Author Contributions

Shishu Huang and Jiancheng Zeng contributed to the study design and supervision. Kai Wang, Xiandi Wang, Lihang Wang, Chuan Luo, and Tianhang Xie contributed to the study design, data extraction and statistical analysis, and drafting of the manuscript. Kai Wang and Zhuhai Li contributed to data extraction and response to reviewers. All authors contributed to the review and revision of the manuscript. All authors read and approved the final manuscript.

## Conflict of Interest Statement

The authors declare that there are no conflicts of interest.

## Supporting information


**Table S1.** Intraclass correlation coefficients (ICC) test showing excellent concordance between both observers.
