# Supplementary material for: The Influence of Screw Positioning on Cage Subsidence in Patients with Oblique Lumbar Interbody Fusion Combined with Anterolateral Fixation
Source: Orthop Surg. 2023 Sep 28;15(12):3263–71. doi: 10.1111/os.13882 (PMC10694007; doi:10.1111/os.13882)
Supplement: Supplementary file 1 — Table S1. Intraclass correlation coefficients (ICC) test showing excellent concordance between both observers. [file OS-15-3263-s001.docx]

| Variable | ICC | 95％CI | p value |
| --- | --- | --- | --- |
| Cage position (mm) | 0.752 | 0.654-0.825 | **＜0.05** |
| CPA (°) | 0.876 | 0.800-0.921 | **＜0.05** |
| SSA (L4) (°) | 0.820 | 0.734-0.878 | **＜0.05** |
| ISA (L5) (°) | 0.851 | 0.765-0.904 | **＜0.05** |
| CA(°) | 0.806 | 0.613-0.892 | **＜0.05** |
| IP (L4) (mm) | 0.858 | 0.796-0.902 | **＜0.05** |
| IP (L5) (mm) | 0.755 | 0.604-0.845 | **＜0.05** |

**[Supplementary Table 1.](https://mc.manuscriptcentral.com/os?DOWNLOAD=TRUE&PARAMS=xik_jqfyU6DUEtnQg6fw6wHegU1VRDiMUBcLFyaULM3VSvVtgEUBGsRKgTTCtKvLXHTdFnPhkZmVj3YYFUZutCMdzmPkMR9mDPkoFdzQ8MSCLn1SKXiwqEcqKgtAPDTyvPzL9kscjVwWg9SNL4aQtdhMqFBw2gqg85DHd24eFNYSMoqveMAxio7YtwmJPuj3Gv5HpDPU)：intraclass correlation coefficients (ICC) test showing excellent concordance between both observers.**
